# Choline as a prevention for Alzheimer’s disease

**DOI:** 10.18632/aging.102849

**Published:** 2020-02-09

**Authors:** Ramon Velazquez, Wendy Winslow, Marc A. Mifflin

**Affiliations:** 1 Arizona State University-Banner Neurodegenerative Disease Research Center at the Biodesign Institute, Arizona State University, Tempe, AZ 85287, USA

**Keywords:** choline, prevention, aging, Alzheimer’s disease, microgliosis

**Figure 1:**
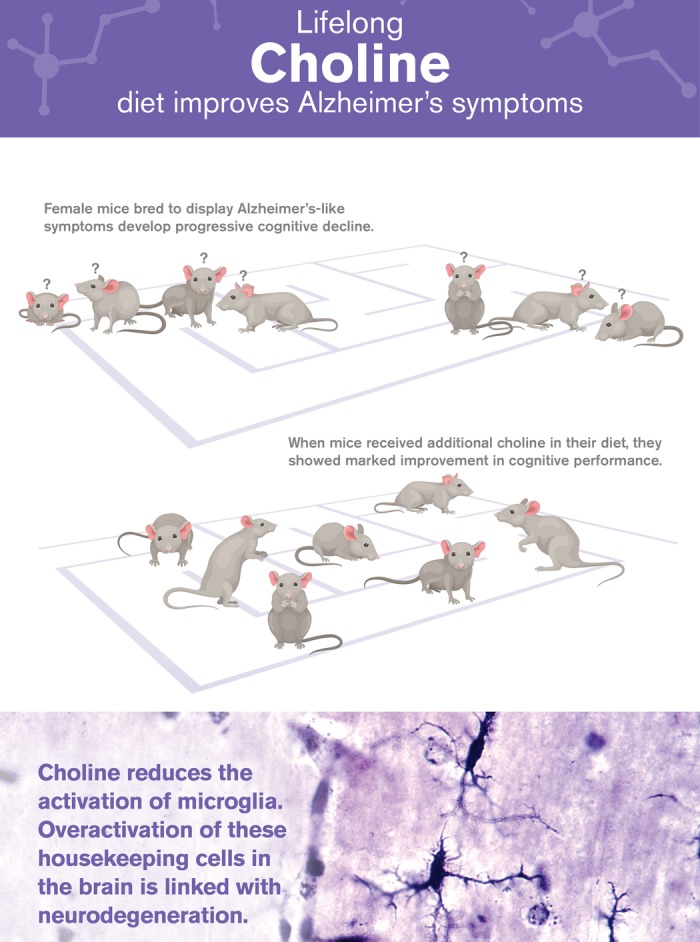
A lifelong regimen of choline (4.5 times the RDI) improved cognitive deficits and reduced activated microglia in female AD mice [3].

Alzheimer’s disease (AD) currently affects 6 million in the U.S. and is projected to afflict 14 million Americans by 2050 [1]. The costs associated with managing AD are expected to exceed $20 trillion in the same time span [1]. The neuropathologies in AD include, Amyloid-β (Aβ) plaques, neurofibrillary tangles, and neuronal loss, which are associated with cognitive impairments [1]. Notably, microglia, the brains resident immune cells, are specialized to rid the brain of deleterious debris. Although microglia keep the brain healthy, if they are overactivated, brain inflammation and neuronal death occurs [2]. To date, no treatments have been developed to effectively slow the progression of AD. A multitude of factors are believed to contribute to the development of the disease, including genetics (e.g. APOE status), age and lifestyle [2]. Interestingly, for reasons that remain unknown, females have an increased risk of developing AD [1). Moreover, studies have identified diet as a significant factor associated with preventing cognitive decline [2]. Collectively, this was the basis of our recent publication that examined the role of lifelong dietary choline supplementation in the APP/PS1 AD mouse model (Figure 1) [3].

Choline is a B-like nutrient that is endogenously produced by the human body. However, endogenous production fails to meet bodily demands. Dietary choline can be found in common foods. In 1998, the U.S. established a recommended daily intake (RDI) of dietary choline for adult women (425mg/day) and adult men (550mg/day). Choline is required to produce acetylcholine, a neurotransmitter responsible for memory, muscle control and mood. It also builds cell membranes and plays a vital role in regulating gene expression. A converging line of evidence indicates that the current RDI may not be optimal for proper brain health and cognition [2–4]. Decades of research have shown that supplementing the maternal (gestation and lactation) diet with choline produces profound benefits on the offspring’s brain health and cognition [5,6]. In fact, studies have demonstrated amelioration of cognitive deficits in mouse models of Down syndrome and AD [5,6]. Remarkably, a very recent study found that maternal choline supplementation (MCS) can produce transgenerational benefits on AD neuropathology, which has profound implications for generations to come [6].

Our recently published work is amongst the first to show benefits of lifelong choline supplementation in a mouse model of AD [3]. The age at which these mice were choline supplemented is equivalent to humans aged 20 thru 60. While our study focused on female mice, a report this year found similar benefits in male mice [4]. These publications establish that both sexes benefit from additional choline [3,5]. Our work identified that a lifelong regimen of choline supplementation protects the brain from AD both by blocking the production of Aβ and by reducing the activation of microglia (Figure 1), which is consistent with the report in males [4]. Interestingly, one study found transgenerational reductions in activated microglia with MCS [6]. The observed reductions in disease-associated microglia, which are present in various neurodegenerative diseases, offer exciting new avenues of research and suggest ways of treating a broad range of disorders, including traumatic brain injuries, multiple sclerosis and Parkinson’s disease.

As of August 2019, AD and other forms of dementia are the leading cause of death in England and Wales [7]. A recent report suggests that the increase cases in the UK may be associated with people not reaching the choline RDI [7]. Achieving the choline RDI can be accomplished by consuming eggs, red meat and poultry, which are some of the food’s richest in choline. Consumption of meat among those aged 19–64 years in the UK has declined by 19g per day over the last 9 years, which may be contributing to a lack of dietary choline. The U.S. has also reported people not reaching the choline RDI, in particular pregnant women [5,8]. The combined evidence on a lack of people reaching the RDI and the benefits of additional choline poses a twofold problem that necessitates awareness and reconsideration of the choline RDI. The same report from the UK suggests that plant-based dieters may lack choline [7]. However, without accurate data on UK intake, it’s impossible to say whether plant-based diets may be contributing to low choline consumption. Additionally, there are many sources of dietary choline for plant-based consumers, such as, soybeans (1/2 cup; 107mg), Brussel sprouts (1/2 cup; 32mg), and toast (1 oz; 51mg). Furthermore, vitamin supplements containing choline are widely available at affordable costs. Thus, it is possible to reach an adequate intake level of choline for those that are on plant-based diets.

The tolerable upper limit (UL) of choline (3,500mg/day) is 8.24 and 6.36 times higher than the RDI for females and males, respectively. Studies in mice identified cognitive benefits with no side effects using 4.5 times the RDI [2, 4). In humans, a recent study found improvements in information processing speed of 13-month-old infants whose mothers were administered additional choline [8]. This is consistent with work on MCS in mice. No study to date has examined whether increasing human choline intake throughout life produces benefits on cognitive aging and/or prevents AD. Before recommending a lifelong regimen of choline supplementation in humans, a controlled clinical trial will be needed to ultimately determine the effectiveness and optimal dosage of choline to prevent or slow the progression of AD. Nonetheless, the current literature creates optimism that choline may be an avenue to ensure a graceful aging process without cognitive decline.
